# The role of sarcopenic obesity for the prediction of prognosis of patients with gastrointestinal cancer

**DOI:** 10.1002/cam4.7452

**Published:** 2024-07-02

**Authors:** Wenqing Chen, Qinggang Yuan, Xiangrui Li, Jiashu Yao, Lihua Yuan, Xiaotian Chen, Bo Gao

**Affiliations:** ^1^ Department of Clinical Nutrition, Nanjing Drum Tower Hospital The Affiliated Hospital of Nanjing University Medical School Nanjing Jiangsu China; ^2^ Department of General Surgery Nanjing Drum Tower Hospital Clinical College of Xuzhou Medical University Nanjing Jiangsu China; ^3^ Department of Interventional Radiology, Nanjing Drum Tower Hospital The Affiliated Hospital of Nanjing University Medical School Nanjing Jiangsu China

**Keywords:** gastrointestinal cancer, nutrition, sarcopenia, sarcopenic obesity

## Abstract

**Background:**

Sarcopenic obesity (SO) in patients with gastrointestinal cancer is associated with a poor prognosis. We aimed to investigate the prognostic impact of SO in patients with gastrointestinal cancer, as well as the diagnostic cut‐off value of SO in patients with gastrointestinal cancer among Chinese population.

**Methods:**

We conducted a consecutive cohort study. Between January 2017 and January 2019, 289 patients diagnosed with gastrointestinal cancer were included in our study. Skeletal muscle area, total fat area, and subcutaneous fat area were measured by CT scan. All patients were followed up for 5 years. Receiver operating characteristic curves (ROC) were adopted to determine the cut‐off values of visceral fat obesity for the prediction of sarcopenia. Based on the cut‐off values, patients with sarcopenia combined with visceral fat obesity were divided into the SO group, and the others were divided into the non‐sarcopenic obesity (NSO) group. Kaplan–Meier curves and univariate and multivariate Cox proportional hazard models were employed to explore the associations of body composition profiles with 5‐year overall survival and disease‐specific survival.

**Results:**

Obtained from Youden's Index for ROC for the prediction of 5‐year survival, skeletal muscle mass index (SMI) ≤40.02 cm^2^/m^2^ with VFA ≥ 126.30 cm^2^ in men and SMI ≤32.05 cm^2^/m^2^ with VFA ≥72.42 cm^2^ in women indicate a risk of poor prognosis in patients diagnosed with gastrointestinal cancer. Patients with SO had poorer 5‐year overall survival (OS) than patients with NSO (6.74% vs. 82.84%, *p* < 0.001), and poorer 5‐year DFS (6.74% vs. 81.82%, *p* < 0.001). In multivariate analysis, we found that the long‐term mortality risk was approximately 13‐fold higher among patients in the SO group compared to those with no conditions.

**Conclusions:**

Preoperative assessment of SO is useful not only for monitoring nutritional status but also for predicting 5‐year OS in gastrointestinal cancer patients.

## Graphical abstract

In this work, we report the first study on combining skeletal muscle mass index (SMI) with VFA and identified the cut‐off value to predict overall prognosis of patients with gastrointestinal cancer among Chinese population in gastrointestinal cancers. This manuscript revealed that sarcopenic obesity can predict the prognosis of patients with gastrointestinal cancer. SMI ≤40.02 cm^2^/m^2^ with VFA ≥126.30 cm^2^ in men and SMI ≤32.05 cm^2^/m^2^ with VFA ≥72.42 cm^2^ in women is a risk factor for poor prognosis in patients with gastrointestinal cancer. This may provide the basis for early prediction, diagnosis, and treatment of sarcopenia obesity in gastrointestinal cancer patients and reducing the risk of adverse outcomes.

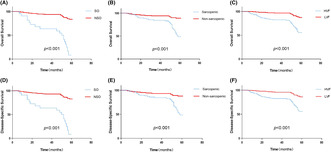

## 1 INTRODUCTION

The global incidence of gastrointestinal cancer has significantly increased in recent years, leading to high mortality and morbidity rates.^1^ The prognosis of gastrointestinal cancer imposes a heavy economic and psychological burden on patients and society.^2^ Nutritional imbalance is a common issue in patients with gastrointestinal cancer and has been reported to be closely associated with poor prognosis such as wound infections, anastomotic leakage, and an increased probability of conversion to open surgery.^3^ Therefore, early nutritional screening and rational nutritional intervention to correct malnutrition in patients with gastrointestinal cancer are crucial.

Sarcopenic obesity (SO), the state of having both sarcopenia and obesity, has been linked to adverse prognosis among patients with gastrointestinal cancer.^4^ Sarcopenia affects approximately 40%–80% of patients with gastrointestinal cancers.^5^ Gastrointestinal cancer mainly affects the digestive tract leading to inadequate dietary intake.^6^, ^7^ Previous research has proved that sarcopenia was an independent risk factor for poor outcome in individuals with various types of cancer and can be used to predict overall survival (OS).^8^ High visceral fat area (VFA) is significantly related to pro‐inflammation and is considered an indicator associated with poorer colon cancer outcomes and a worse prognosis in colorectal,^9^ esophageal,^10^ and gastric cancers.^11^

The nutritional status of patients was often easily neglected by clinicians, leading to delayed provision of necessary nutritional support. Particularly in patients with SO, without a computed tomography (CT) scan, these patients are often under‐diagnosed as not at nutritional risk because they do not have significant short‐term weight changes and visible signs of wasting. Malnutrition compromises patient outcomes, and failure to provide timely nutritional intervention can have serious consequences. Therefore, early nutritional screening and timely detection of SO patients is essential in helping them reverse malnutrition.^12^

Previous studies primarily concentrated on Caucasians, while the human body composition of different populations varies widely. Currently, there is no consistent diagnostic cut‐off value for skeletal muscle mass index (SMI) in the Chinese population, and the diagnostic cut‐off values for visceral fat obesity differ among different populations. In this study, we aim to investigate the diagnostic cut‐off value of SO in patients with gastrointestinal cancer in the Chinese population and explore the prognostic effects of SO in patients with gastrointestinal cancer.

## 2 MATERIALS AND METHODS

### 2.1 Participants

This trial recruited gastrointestinal cancer patients from the Affiliated Drum Tower Hospital of Nanjing University Medical College in Nanjing, China, between January 2017 and January 2019. Inclusion criteria were as follows: (1) all patients were newly diagnosed and did not receive surgery, chemotherapy, radiation, or neoadjuvant therapy; (2) radical surgery in our hospital; (3) basic information, medical history, auxiliary examination, nutritional indexes, and other data were complete. Exclusion criteria were as follows: (1) under the age of 18; (2) severe edema, such as pleural effusion or ascites, cardiac or kidney failure, cirrhosis, and the use of diuretic drugs; (3) patients with distant metastases; (4) refuse to participate or unable to cooperate, use of lipid‐regulating drugs, protein supplements, etc., in the last 3 months. This study was approved by the Clinical Research Ethics Committee of Nanjing Drum Tower Hospital. Written informed consent was obtained from all participants. Among the eligible patients, 289 patients with gastrointestinal cancers participated in the final study.

### 2.2 CT anthropometric measurements

All participants underwent CT scans within the first 24 h after admission. The methods for analyzing body composition using CT scanning were adapted from previously published protocols.^13^ Skeletal muscle area, measured at the level of the third lumbar vertebra (L3), is an accurate surrogate for total skeletal muscle mass^14^ and was selected for analysis using Matlab software (MathWorks, USA). The skeletal muscle area was manually sketched and segmented using Hounsfield units (HU) ranging from −29 to 150 to represent skeletal muscle tissue. The radiation attenuation was expressed as the mean HU of the compartment. The SMI was defined as the area of skeletal muscle mass, usually calculated as the area relative to the patient's height (SMI = skeletal muscle mass at L3 [cm^2^]/square of body height [m^2^]), which is then used to assess patients for sarcopenia. The VFA was measured at the level of the umbilical slice level on the CT image, while the subcutaneous fat area (SCFA) was measured on CT slices at the same location. The VFA was manually outlined and calculated using MATLAB software (MathWorks, Massachusetts, USA) and was calculated within the range of HU from −150 to −50. CT measurements were performed independently by two doctors who have received medical training and worked independently from one another. They were blinded to the patients' personal information.

### 2.3 Data collection and follow‐up

The patients' demographic details including age, height, weight, education level, smoking, and drinking history, etc., were collected from the medical card. Tumor stage classification followed the criteria set by the American Joint Committee on Cancer,^15^ devoid of any subjective evaluations. The WHO/Eastern Cooperative Oncology Group scale was used to grade the performance status (0 = normal performance, 4 = bed‐bound). Tumor markers were assessed upon admission, with parameters including carcinoembryonic antigen, carbohydrate antigen 125, carbohydrate antigen 199, carbohydrate antigen 724, and carbohydrate antigen 242. A fasting blood sample was collected during the first admission for laboratory testing to observe the impact of covariates on the study outcomes.

The follow‐up time was defined as from the surgery date up to the death or the end of follow‐up (August 31, 2022), whichever came first. Patients were monitored via telephone every 3 months for 2 years and every 6 months thereafter for up to 5 years under an established follow‐up program to gather relevant information on clinical outcomes. The primary endpoints were OS and disease‐free survival (DFS). The secondary endpoint was medically confirmed recurrence.

### 2.4 Statistical analysis

Statistical analyses were performed using SPSS software (version 22.0, IBM, USA) and graphs were created with Prism version 8.0.2 (GraphPad). Continuous data were expressed as either the mean ± standard deviation or median (25th percentile and 75th percentile). The Mann–Whitney *U*‐test or independent Student *t*‐test were used to compare continuous variables. Categorical variables were analyzed with the *χ* ^2^ test. Receiver operating characteristic curves (ROC) were utilized to determine the cut‐off values for both visceral fat obesity and sarcopenia. The ideal cut‐point was established using the Youden Index (Youden Index = sensitivity + specificity −1). According to the cut‐off values of the body composition profiles, the population was divided into two groups: SO and non‐sarcopenic obesity (NSO) groups. Kaplan–Meier method and log‐rank test were used to investigate the relationships between body composition profiles and both the 5‐year OS and DFS rates. Univariable and multivariate regression was undertaken using Cox proportional hazards regression models to calculate hazard ratios (HRs) and 95% confidence intervals (CIs), and the forward LR stepwise selection method was used to assess the potential interaction effects of variables in the multivariate model. A two‐sided *p* < 0.05 was considered statistically significant.

## 3 RESULTS

### 3.1 Baseline

**FIGURE 1:**
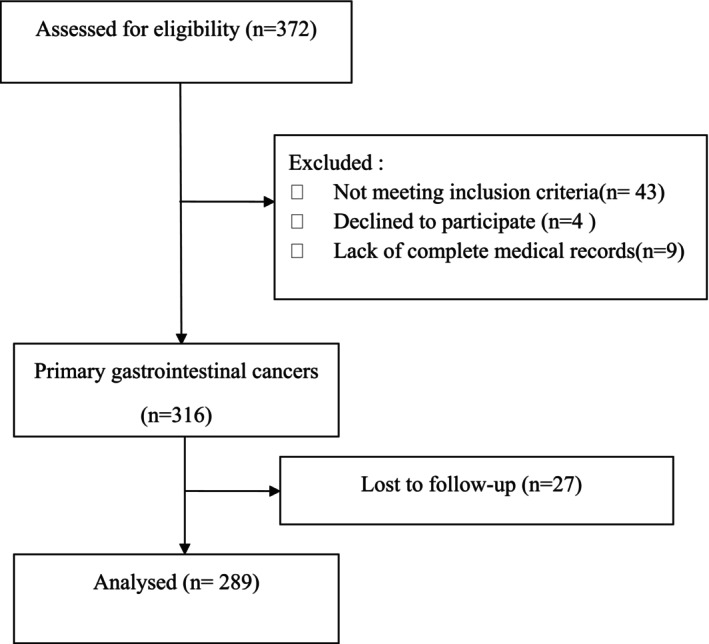
Flow diagram of the research.

Between January 2017 and January 2019, 372 patients with gastrointestinal cancer were screened for eligibility and 289 of them were included in this study (Figure 1). The baseline characteristics of the patients were presented in Table 1.

**TABLE 1:** Baseline characteristics.

	Overall *N* = 289	NSO *n* = 267	SO *n* = 22	*p* value
Age (year)	67.6 ± 11.4	67.5 ± 11.5	69.4 ± 9.8	0.440
Male (*n*, %)	183 (63.32)	171 (64.04)	12 (54.55)	0.374
Height (cm)	165.2 ± 6.8	165.1 ± 6.8	166.2 ± 8.0	0.451
Weight (kg)	64.2 ± 9.9	64.1 ± 9.7	66.2 ± 12.4	0.438
BMI (kg/m^2^)	23.51 ± 3.11	23.49 ± 3.12	23.79 ± 2.97	0.668
Smoking history (*n*, %)	38 (13.1)	34 (12.7)	4 (18.2)	0.467
Drinking history (*n*, %)	13 (4.5)	12 (4.5)	1 (4.5)	0.991
Tumor location				0.597
Gastric cancer	116 (40.14)	106 (39.70)	10 (45.45)	
Colorectal cancer	173 (59.86)	161 (60.30)	12 (54.55)	
Tumor stage (AJCC)				0.930
I	74 (25.5)	62 (23.7)	4 (21.1)	
II	122 (43.4)	114 (43.5)	8 (42.1)	
III	93 (33.1)	86 (32.8)	7 (36.8)	
ECOG	0.20 ± 0.47	0.20 ± 0.46	0.18 ± 0.50	0.843
Laboratory parameters				
WBC (×10^9^/L)	5.98 ± 2.05	5.98 ± 2.09	5.96 ± 1.52	0.958
Hb (g/L)	121.80 ± 23.8	122.5 ± 23.4	112.4 ± 26.9	0.055
Platelet (×10^9^/L)	205.5 (167.0, 253.5)	205.0 (168.0, 251.0)	214.5 (144.0, 281)	0.419
Alb (g/L)	39.20 ± 3.3	39.2 ± 3.3	38.8 ± 3.0	0.555
CRP (mg/L)	3.50 (2.40, 7.30)	3.50 (2.40, 7.20)	3.65 (2.60, 14.70)	0.741
CRE	64.08 ± 22.07	64.01 ± 22.36	62.40 ± 12.03	0.503
eGFR	116.96 ± 29.61	118.46 ± 28.16	110.58 ± 18.55	0.874
CEA (ng/mL)				0.580
≤5 (*n*, %)	236 (81.7)	219 (82.0)	17 (77.3%)	
>5 (*n*, %)	53 (18.3)	48 (18.0)	5 (22.7%)	
CA199 (U/mL)				0.186
≤37 (*n*, %)	260 (90)	242 (90.6)	18 (81.8)	
>37 (*n*, %)	29 (10)	25 (9.4)	4 (18.2)	
Follow‐up (month)	52 (48, 58)	52 (48.5, 59)	51 (19, 54)	0.034*
DFS (month)	52 (48, 58)	52 (48, 59)	51 (19, 54)	0.042*

*Note*: Data are presented as mean ± standard deviation or median (quartile 1, quartile 3).

Abbreviations: Alb, albumin; BMI, body mass index; Ca199, carbohydrate antigen 199; CEA, carcinoembryonic antigen; CRE, creatinine; CRP, C‐reactive protein; DFS, disease‐free survival; ECOG, Eastern Cooperative Oncology Group; eGFR, esti mated glomerular filtration rate; Hb, hemoglobin; WBC, white blood cells.

* *p* < 0.05.

There were 183 males and 106 females enrolled in this study, their mean age was 67.6 ± 11.4 years old. A total of 116 tumors (40.1%) were gastric cancer and 173 tumors (59.9%) were colorectal cancer. Patients were followed up clinically for a median of 52 (IQR: 48, 58) months.

### 3.2 Cut‐off values for visceral fat obesity and sarcopenia

**FIGURE 2:**
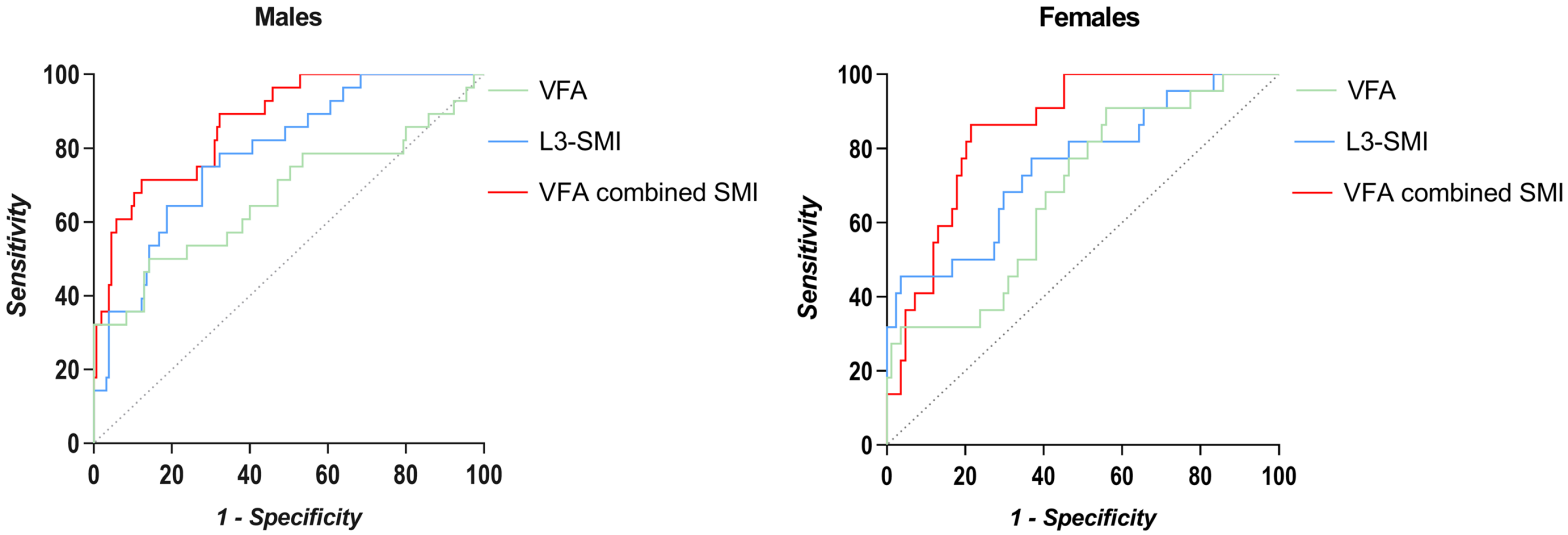
The ROC curves of body composition profiles for the prediction of 5‐year mortality in males and females. L3‐SMI, L3‐ skeletal muscle index, VFA, visceral fat area.

Utilizing ROC curves to predict 5‐year OS and Youden's Index, we identified the sex‐specific cut‐off values for visceral fat obesity and sarcopenia (Figure 2). Sarcopenia was defined as SMI ≤40.02cm^2^/m^2^ in men and SMI ≤32.05 cm^2^/m^2^ in women. Visceral fat obesity was defined as VFA ≥126.30 cm^2^ in men and VFA ≥72.42 cm^2^ in women in the present study (Table 3). Sarcopenia patients accounted for 27.34% of the study population and 35.99% of patients were visceral fat obese (Table 2).

**TABLE 2:** Body composition ssprofiles in patients with gastrointestinal cancer.

Variable	Overall *N* = 289	NSO *n* = 267	SO *n* = 22	*p* value
Skeletal muscle area	112.58 ± 22.90	114.18 ± 22.44	93.21 ± 19.81	0.000*
VF area (cm^2^)	88.18 ± 47.68	83.39 ± 44.63	146.35 ± 45.91	0.000*
SC area (cm^2^)	114.48 ± 55.79	113.73 ± 57.14	123.64 ± 35.09	0.119
SMI (cm^2^/m^2^)	41.07 ± 7.01	41.71 ± 6.81	33.36 ± 4.31	0.000*
Sarcopenia (*n*, %)	79 (27.34%)	57 (21.35%)	22 (100%)	0.000*
Visceral fat obesity (*n*, %)	104 (35.99%)	82 (30.71%)	22 (100%)	0.000*

*Note*: Data are presented as mean ± standard deviation.

Abbreviations: SC, subcutaneous fat; SMI, skeletal muscle index; VF, visceral fat.

* *p* < 0.001.

**TABLE 3:** Diagnostic accuracy of body composition profiles to predict overall survival.

	Males						Females					
	Cut‐off value	AUC (95%CI)	Sensitivity (%)	Specificity (%)	Youden index	*p* value	Cut‐off value	AUC (95%CI)	Sensitivity (%)	Specificity (%)	Youden index	*p* value
SMI (cm^2^/m^2^)	40.02	0.787 (0.701, 0.872)	0.750	0.723	0.473	0.000*	32.05	0.75 (0.634, 0.873)	45.45%	96.43%	0.419	0.000*
VFA (cm^2^)	126.30	0.669 (0.540, 0.798)	0.500	0.858	0.358	0.004*	72.42	0.68 (0.5629, 0.8050)	90.91%	44.05%	0.350	0.008*

*Note*: Receiver operating characteristic curve to evaluate the cut‐off for body composition profiles.

Abbreviations: SC, subcutaneous fat; SMI, skeletal muscle index; VF, visceral fat.

* *p* <0.05 or *p* < 0.001.

**TABLE 4:** Cox hazard regression analysis for factors associated with 5‐year overall survival.

	Univariable analysis			Multivariate analysis		
Variable	*B*	HR (95%CI)	*p* value	*B*	HR (95%CI)	*p* value
Presence of SO	2.187	8.904 (4.970, 15.954)	0.000*	2.605	13.529 (7.064, 25.912)	0.000*
Tumor stage						
1	Reference			Reference		
2	0.946	2.576 (1.038, 6.393)	0.041*	0.844	2.325 (0.881, 6.133)	0.088
3	1.446	4.247 (1.724, 10.461)	0.002*	1.579	4.849 (1.850, 12.709)	0.001*
Alb	−0.117	0.890 (0.823, 0.962)	0.003*	−0.107	0.899 (0.815, 0.991)	0.032*
Hb	−0.016	0.984 (0.973, 0.995)	0.004*			
PLT	0.004	1.004 (1.000, 1.000)	0.033*			
CA199	0.005	1.005 (1.000, 1.000)	0.048*			

Abbreviations: Alb, albumin; BMI, body mass index; SMI, skeletal muscle index; VF, visceral fat.

* *p* < 0.05 or *p* < 0.001.

**TABLE 5:** Cox hazard regression analysis for factors associated with 5‐year disease‐specific survival.

	Univariable analysis			Multivariate analysis		
Variable	*B*	HR (95%CI)	*p* value	*B*	HR (95%CI)	*p* value
Presence of SO	2.187	8.904 (4.970, 15.954)	0.000*	2.594	13.387 (6. 991, 25.634)	0.000*
Tumor stage						
1	Reference			Reference		
2	0.962	2.576 (1.054, 6.491)	0.038*	0.860	2.362 (0.896, 6.229)	0.082
3	1.463	4.321 (1.753, 10.648)	0.001*	1.592	4.913 (1.873, 12.888)	0.001*
Alb	−0.117	0.890 (0.823, 0.961)	0.003*	−0.106	0.899 (0.816, 0.991)	0.032*
Hb	−0.016	0.984 (0.973, 0.995)	0.004*			
PLT	0.004	1.004 (1.000, 1.007)	0.038*			
CA199	0.005	1.005 (1.000, 1.011)	0.048*			

Abbreviations: Alb, albumin; BMI, body mass index; SMI, skeletal muscle index; VF, visceral fat.

* *p* < 0.05 or *p* < 0.001.

Based on the cut‐off values of the body composition profiles, patients with sarcopenia combined with visceral fat obesity were divided into the SO group and the others were divided into the NSO group. In this study, 22 individuals (7.61%) were identified with SO, 79 (27.34%) were diagnosed with sarcopenia, and 104 patients (35.99%) experienced visceral fat obesity (Table 2). There were no statistically significant differences were found in the demographic and clinic opathological data between the SO and NSO groups (Table 1). The median duration of follow‐up within the SO group was significantly lower than that of the NSO group (51 (IQR: 19, 54) vs. 52(IQR: 48.5, 59), *p* = 0.034). The DFS was also significantly lower in the SO group compared to the NSO group (*p* = 0.034).

### 3.3 Associations of body composition profiles with gastrointestinal cancer outcomes

**FIGURE 3:**
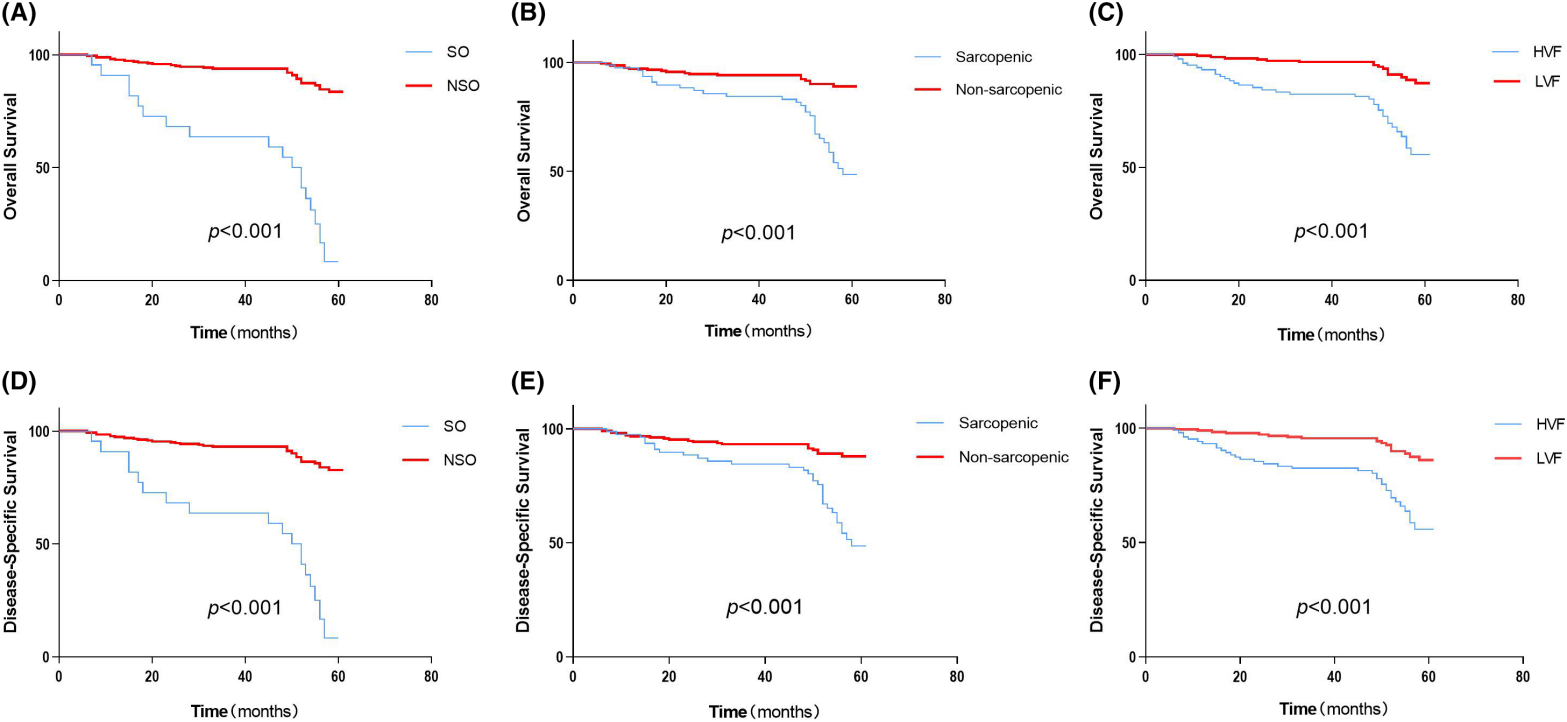
(A) Kaplan–Meier curves for 5‐year overall survival in patients with and without sarcopenic obesity (SO). (B) Kaplan–Meier curves for 5‐year overall survival in patients with and without sarcopenia. (C) Kaplan–Meier curves for 5‐year overall survival in patients with and without visceral fat obesity. (D) Kaplan–Meier curves for 5‐year disease‐specific survival in patients with and without SO. (E) Kaplan–Meier curves for 5‐year disease‐specific survival in patients with and without sarcopenic. (F) Kaplan–Meier curves for 5‐year disease‐specific survival in patients with and without SO. HVF, high visceral fat; LVF, low visceral fat; NSO, non‐sarcopenic obesity; SO, sarcopenic obesity.

The body composition profiles were significantly linked to with 5‐year OS and DFS. The 5‐year OS rate (14.76% vs. 24.05%, *p* < 0.001) and 5‐year DFS rate (14.76% vs. 26.58%, *p* < 0.001) in the sarcopenic group were significantly lower than that in the non‐sarcopenic group (Figure 3B,E). The 5‐year OS rate (37.84% vs. 15.38%, *p* < 0.001) and the 5‐year DFS rate (37.84% vs. 15.38%) in the visceral fat obesity group were both significantly higher than that in the non‐visceral fat obesity group (Figure 3C,F). Patients with SO had poorer 5‐year OS than patients with NSO (6.74% vs. 82.84%, *p* < 0.001), and SO patients also had poorer 5‐year DFS than NSO patients (6.74% vs. 81.82%, *p* < 0.001) (Figure 3A,D).

### 3.4 Univariate and multivariate Cox regression of body composition profiles with OS and DFS for gastrointestinal cancer

Univariable analysis revealed that presence of SO was significantly associated with poor OS (HR 8.904, 95% CI 4.970–15.954, *p* = 0.000). In addition, low Alb (HR 0.890, 95% CI 0.823–0.962, *p* = 0.003), low Hb (HR 0.984, 95% CI 0.973–0.995, *p* = 0.004), were significantly associated with poor OS (Table 4). Factors associated with DFS were shown in Table 5. In the multivariate regression model containing the statistical significantly variables, we found that presence of SO (HR 13.529, 95% CI 7.064–25.912, *p* = 0.000), was risk factor for OS, tumor stage III was associated with a 4.894‐fold increase overall risk of death compared to patients with tumor staged I (HR 4.849, 95% CI 1.850–12.709, *p* = 0.027), Alb (HR 0.899, 95% CI 0.815–0.991, *p* = 0.032) was protective factors for OS (Table 4). Multivariate analysis identified the presence of SO (HR 13.387, 95% CI 6. 991–25.634, *p* = 0.000) and advanced tumor stage as risk factors for DFS. Patients with tumors stage III exhibited a 4.913‐fold increased risk of overall disease‐free mortality compared to stage I (HR 4.913, 95% CI 1.873–12.888, *p* = 0.028), In contrast, Alb (HR 0.899, 95% CI 0.816–0.991, *p* = 0.032) was a protective factor for DFS (Table 5).

## 4 DISCUSSION

In this study, it was determined that SO can predict the prognosis of patients with gastrointestinal cancer. Patients with gastrointestinal cancer who had SO experienced lower 5‐year OS (HR 13.529, 95% CI 7.064–25.912, *p* = 0.000) as well as disease‐specific survival rates (HR 13.387, 95% CI 6.991–25.634, *p* = 0.000). The presence of SO, lower Alb, and advanced tumor stage were identified as risk factors for OS and DFS. Moreover, this is the first study to combine SMI with VFA and identified the cut‐off value to predict overall prognosis of patients with gastrointestinal cancer among Chinese population. In males, SO was diagnosed if SMI ≤40.02 cm^2^/m^2^ and VFA ≥126.30 cm^2^, and in females, SO was diagnosed if SMI ≤32.05 cm^2^/m^2^ and VFA ≥72.42 cm^2^.

SO is a state characterized by decreased skeletal muscle mass and visceral fat mass.^16^ Our results was in consistency with previous conclusions that patients with cancer who develop SO tend to have a poorer prognosis.^17^, ^18^ A meta‐analysis conducted by Wang et al. on 8729 patients who underwent surgery for gastrointestinal cancer revealed that SO was associated with an increased risk of postoperative complications and a lower survival rate in patients with gastrointestinal cancer.^19^ Several studies have found that the presence of SO in cancer patients is correlated with negative outcomes. Kobayashi et al. discovered that preoperative SO was a significant risk factor for mortality and HCC recurrence following hepatectomy for HCC.^20^ Kim et al. noted that SO was an independent risk factor for increased mortality in patients suffering from gastric cancer.^21^

In our study, both of VFA and SMI were significantly associated with OS and DFS, which was corroborated with previously reported studies. Gastrointestinal cancers, such as stomach and colorectal cancer, have a high incidence of sarcopenia.^22^ Sarcopenia is characterized as a clinical indication of the cancer cachexia syndrome^23^ and may lead to the loss of muscle strength, impaired immune function and reduced tolerance to cancer treatments such as chemotherapy or surgery. These factors contribute to an increased risk of complications in patients with gastrointestinal cancer.^23^ Visceral fat obesity is associated with the development of chronic inflammation and insulin resistance, both of which are critical in both tumor development and further progression.^24^ It has been documented that visceral fat obesity is a risk factor for different cancer complications.^25^, ^26^ Studies have increasingly recognized the importance of muscular fatty infiltrations for the loss of skeletal muscle function.^27^ Fat accumulation in muscle tissue initiates a pro‐inflammatory response and oxidative stress, consequently resulting in mitochondrial dysfunction, impaired insulin signaling, and subsequent muscle atrophy.^28^ Sarcopenia and visceral fat obesity are both clinically detectable and diagnosable. To combine skeletal muscle marker with visceral fat marker could comprehensively reflect body immune system and insulin resistance. Thus, patients with SO display an increased susceptibility to tumor development and an unfavorable prognosis. In previous studies of SO cut‐off values, the study populations were selected from groups with heterogeneous characteristics. Furthermore, the majority of studies used existing data from healthy populations or thresholds from disease‐specific populations for diagnosis.^29^, ^30^, ^31^ However, the absence of a universally applicable cut‐off value for different diseases remains a contentious issue due to the limited transferability of utilization among heterogeneous patient cohorts in different cancer patient populations, especially in Chinese population. In our study, we performed ROC analysis on our own data to find a Chinese specific cut‐off value for the diagnostic threshold that is appropriate for the population of patients with gastrointestinal tumors in Eastern China. This highlights the need for further researches to better understand the underlying mechanisms behind this condition.

Sarcopenia is generally linked to weight loss, while visceral fat obesity is associated with weight gain. However, patients with SO who do not undergo a CT scan are often misdiagnosed as not being at nutritional risk, as they lack significant short‐term weight changes and visible signs of wasting. Without appropriate nutritional interventions, malnutrition can have a detrimental effect on patient outcomes and result in severe consequences. The implementation of appropriate testing can facilitate the achievement of early detection, early diagnosis, and timely early nutritional intervention, thereby promoting the likelihood of improved clinical outcome results. Consequently, the timely identification and screening of malnourished patients with SO is imperative to enable them to reverse their malnutrition.^12^ Our study has revealed that SO is associated with a poor prognosis in gastrointestinal cancers. It is recommended that clinicians raise awareness of this indicator in clinical practice in order to facilitate the implementation of early nutritional interventions for patients with SO, and thereby enhance patient prognosis.

Interestingly, there were no statistically significant differences in demographic and clinicopathological data between the SO and NSO groups in our study. This phenomenon could elucidate why BMI is an unreliable prognostic indicator for cancer surgery, as it fails to account for disparities in fat or muscle mass distribution^32^ This observation has also been referred to as the obesity paradox in cancer, as high sex‐specific BMI levels do not always correlate with poor cancer prognosis.^10^ Combined with a state of visceral fat obesity, this can obscure reduced muscle mass due to malnutrition. SO may be influenced by factors beyond conventional risk factors in cancer patients, such as age, gender, and body mass index. Several studies have described that higher subcutaneous fat area has a protective association with the deleterious inflammatory outcomes associated with visceral fat.^33^, ^34^ Many patients with normal BMI might actually harbor SO to various degrees, before it progresses to full‐blown severe sarcopenia.^16^ It is theoretically possible that tumor staging may affect the composition of human body components.^35^, ^36^ However, there may be no distributional differences in this study due to the relatively small sample size and other issues.

Our study has some limitations. First, being a single‐center study, it may have a selection bias. Large‐sample multicenter studies are required to validate our conclusions, as well as a development cohort and a validation cohort. Our future work will focus on expanding the sample size and incorporating more relevant influencing factors affecting prognosis. Furthermore, we will obtain patients' consent to obtain blood samples during the study period to improve the genomics analysis. Additionally, using only the cross‐sectional skeletal muscle area for SMI calculation may have potentially led to underestimated prevalence estimates. More research is required to assess the impact of muscle strength on the survival of patients with gastrointestinal cancer. This establishes a more accurate, stable, and reliable prediction model that can be applied to clinical practice. Finally, specific follow‐up on nutritional support was not included, making it impossible to determine whether changes in nutritional status have an effect on SO. This highlights the necessity for further research to better comprehend the potential influence of nutrition‐related factors behind this condition.

## 5 CONCLUSION

Patients with SO had worse 5‐year OS and DFS. Sarcopenia is diagnosed when SMI ≤40.02 cm^2^/m^2^ in men and SMI ≤32.05 cm^2^/m^2^ in women. Visceral fat obesity was diagnosed when VFA ≥126.30 cm^2^ in men and VFA ≥72.42 cm^2^ in women. Preoperative assessment of SO is beneficial not only for monitoring nutritional status but also for predicting long‐term OS in patients with gastrointestinal cancer. Early intervention for SO patients is helpful to improve the clinical prognosis of patients, bolster their quality of life, cut down on medical expenses and recurrent hospitalization, and assuage the medical and economic burden on the community.

## AUTHOR CONTRIBUTIONS

**Wenqing Chen:** Data curation (lead); formal analysis (lead); investigation (lead); methodology (equal); writing – original draft (lead). **Qinggang Yuan:** Data curation (equal); formal analysis (lead); methodology (equal); software (equal). **Xiangrui Li:** Writing – review and editing (equal). **Jiashu Yao:** Data curation (equal); formal analysis (equal). **Lihua Yuan:** Methodology (lead); resources (equal); software (equal). **Xiaotian Chen:** Funding acquisition (equal); resources (supporting); supervision (equal); writing – review and editing (equal). **Bo Gao:** Conceptualization (lead); data curation (equal); funding acquisition (lead); methodology (lead); resources (supporting); writing – review and editing (equal).

## FUNDING INFORMATION

The research was funded by the New Medical Technology of Nanjing Drum Tower Hospital (XJSFZLX202127, XJSFZLX202363), the Institute of Hospital Management, Nanjing University (NDYG2023038), Japan China Sasakawa Medical Fellowship and Science Foundation of Nanjing Drum Tower Hospital (2022‐yxzx‐yx‐05).

## CONFLICT OF INTEREST STATEMENT

The authors declare no competing interests.

## ETHICS STATEMENT

The observational study was in accordance with the ethics principles of the Declaration of Helsinki and was approved by the Clinical Research Ethics Committee of Nanjing Drum Tower Hospital.

## Data Availability

The data underlying this article will be shared upon reasonable request from the corresponding author.

## References

[1] Youn NS , Park BJ , Nam JH , et al. Association of current helicobacter pylori infection and metabolic factors with gastric cancer in 35,519 subjects: a cross‐sectional study. United European Gastroenterol J. 2019;7(2):287‐296. 10.1177/2050640618819402 PMC6498802 31080613

[2] Testa U , Pelosi E , Castelli G . Colorectal cancer: genetic abnormalities, tumor progression, tumor heterogeneity, clonal evolution and tumor‐initiating cells. Med Sci (Basel). 2018;6(2):31. 29652830 10.3390/medsci6020031 PMC6024750

[3] Kuwada K , Kuroda S , Kikuchi S , et al. Clinical impact of sarcopenia on gastric cancer. Anticancer Res. 2019;39(5):2241‐2249. 31092415 10.21873/anticanres.13340

[4] Saino Y , Kawase F , Nagano A , et al. Diagnosis and prevalence of sarcopenic obesity in patients with colorectal cancer: a scoping review. Clin Nutr. 2023;42(9):1595‐1601. 37480796 10.1016/j.clnu.2023.06.025

[5] Chen LK , Woo J , Assantachai P , et al. Asian working group for sarcopenia: 2019 consensus update on sarcopenia diagnosis and treatment. J Am Med Dir Assoc. 2020;21(3):300‐307. 32033882 10.1016/j.jamda.2019.12.012

[6] Bye A , Jordhoy MS , Skjegstad G , et al. Symptoms in advanced pancreatic cancer are of importance for energy intake. Support Care Cancer. 2013;21(1):219‐227. 22684989 10.1007/s00520-012-1514-8

[7] Blum D , Omlin A , Baracos VE , et al. Cancer cachexia: a systematic literature review of items and domains associated with involuntary weight loss in cancer. Crit Rev Oncol Hematol. 2011;80(1):114‐144. 21216616 10.1016/j.critrevonc.2010.10.004

[8] Choi MH , Oh SN , Lee IK , Oh ST , Won DD . Sarcopenia is negatively associated with long‐term outcomes in locally advanced rectal cancer. J Cachexia Sarcopenia Muscle. 2018;9(1):53‐59. 28849630 10.1002/jcsm.12234 PMC5803619

[9] Malietzis G , Aziz O , Bagnall NM , Johns N , Fearon KC , Jenkins JT . The role of body composition evaluation by computerized tomography in determining colorectal cancer treatment outcomes: a systematic review. Eur J Surg Oncol. 2015;41(2):186‐196. 25468746 10.1016/j.ejso.2014.10.056

[10] Lennon H , Sperrin M , Badrick E , Renehan AG . The obesity paradox in cancer: a review. Curr Oncol Rep. 2016;18(9):56. 27475805 10.1007/s11912-016-0539-4 PMC4967417

[11] Donohoe CL , Doyle SL , Reynolds JV . Visceral adiposity, insulin resistance and cancer risk. Diabetol Metab Syndr. 2011;3:12. 21696633 10.1186/1758-5996-3-12 PMC3145556

[12] Reginster JY , Cooper C , Rizzoli R , et al. Recommendations for the conduct of clinical trials for drugs to treat or prevent sarcopenia. Aging Clin Exp Res. 2016;28(1):47‐58. 26717937 10.1007/s40520-015-0517-y PMC4768478

[13] Black D , Mackay C , Ramsay G , et al. Prognostic value of computed tomography: measured parameters of body composition in primary operable gastrointestinal cancers. Ann Surg Oncol. 2017;24(8):2241‐2251. 28324283 10.1245/s10434-017-5829-z PMC5491683

[14] van Vugt JL , Levolger S , Gharbharan A , et al. A comparative study of software programmes for cross‐sectional skeletal muscle and adipose tissue measurements on abdominal computed tomography scans of rectal cancer patients. J Cachexia Sarcopenia Muscle. 2017;8(2):285‐297. 27897414 10.1002/jcsm.12158 PMC5697014

[15] Deng J , Zhang R , Pan Y , et al. Comparison of the staging of regional lymph nodes using the sixth and seventh editions of the tumor‐node‐metastasis (TNM) classification system for the evaluation of overall survival in gastric cancer patients: findings of a case‐control analysis involving a single institution in China. Surgery. 2014;156(1):64‐74. 24929759 10.1016/j.surg.2014.03.020

[16] Li CW , Yu K , Shyh‐Chang N , et al. Pathogenesis of sarcopenia and the relationship with fat mass: descriptive review. J Cachexia Sarcopenia Muscle. 2022;13(2):781‐794. 35106971 10.1002/jcsm.12901 PMC8977978

[17] Okumura S , Kaido T , Hamaguchi Y , et al. Visceral adiposity and sarcopenic visceral obesity are associated with poor prognosis after resection of pancreatic cancer. Ann Surg Oncol. 2017;24(12):3732‐3740. 28871520 10.1245/s10434-017-6077-y

[18] Kocher NJ , Jafri S , Balabhadra S , et al. Is sarcopenia and sarcopenic obesity associated with clinical and pathological outcomes in patients undergoing radical nephroureterectomy? Urol Oncol. 2018;36(4):117‐156. 10.1016/j.urolonc.2017.12.004 29276063

[19] Wang P , Wang S , Ma Y , et al. Sarcopenic obesity and therapeutic outcomes in gastrointestinal surgical oncology: a meta‐analysis. Front Nutr. 2022;9:921817. 35938099 10.3389/fnut.2022.921817 PMC9355157

[20] Kobayashi A , Kaido T , Hamaguchi Y , et al. Impact of sarcopenic obesity on outcomes in patients undergoing hepatectomy for hepatocellular carcinoma. Ann Surg. 2019;269(5):924‐931. 29064889 10.1097/SLA.0000000000002555

[21] Kim J , Han SH , Kim HI . Detection of sarcopenic obesity and prediction of long‐term survival in patients with gastric cancer using preoperative computed tomography and machine learning. J Surg Oncol. 2021;124(8):1347‐1355. 34490899 10.1002/jso.26668 PMC9290491

[22] Bozzetti F . Nutritional interventions in elderly gastrointestinal cancer patients: the evidence from randomized controlled trials. Support Care Cancer. 2019;27(3):721‐727. 30413927 10.1007/s00520-018-4532-3

[23] Ryan AM , Power DG , Daly L , Cushen SJ , Ní Bhuachalla Ē , Prado CM . Cancer‐associated malnutrition, cachexia and sarcopenia: the skeleton in the hospital closet 40 years later. Proc Nutr Soc. 2016;75(2):199‐211. 26786393 10.1017/S002966511500419X

[24] Doyle SL , Donohoe CL , Lysaght J , Reynolds JV . Visceral obesity, metabolic syndrome, insulin resistance and cancer. Proc Nutr Soc. 2012;71(1):181‐189. 22051112 10.1017/S002966511100320X

[25] Takeuchi M , Ishii K , Seki H , et al. Excessive visceral fat area as a risk factor for early postoperative complications of total gastrectomy for gastric cancer: a retrospective cohort study. BMC Surg. 2016;16(1):54. 27494994 10.1186/s12893-016-0168-8 PMC4974690

[26] Zhang Y , Wang JP , Wang XL , et al. Computed tomography‐quantified body composition predicts short‐term outcomes after gastrectomy in gastric cancer. Curr Oncol. 2018;25(5):e411‐e422. 30464692 10.3747/co.25.4014 PMC6209549

[27] Brioche T , Pagano AF , Py G , Chopard A . Muscle wasting and aging: experimental models, fatty infiltrations, and prevention. Mol Asp Med. 2016;50:56‐87. 10.1016/j.mam.2016.04.006 27106402

[28] Hong SH , Choi KM . Sarcopenic obesity, insulin resistance, and their implications in cardiovascular and metabolic consequences. Int J Mol Sci. 2020;21(2):494. 31941015 10.3390/ijms21020494 PMC7013734

[29] Boparai G , Kedia S , Kandasamy D , et al. Combination of sarcopenia and high visceral fat predict poor outcomes in patients with Crohn's disease. Eur J Clin Nutr. 2021;75(10):1491‐1498. 33531636 10.1038/s41430-021-00857-x

[30] van der Werf A , Langius J , de van der Schueren M , et al. Percentiles for skeletal muscle index, area and radiation attenuation based on computed tomography imaging in a healthy Caucasian population. Eur J Clin Nutr. 2018;72(2):288‐296. 29242526 10.1038/s41430-017-0034-5 PMC5842880

[31] Park JW , Chang SY , Lim JS , et al. Impact of visceral fat on survival and metastasis of stage III colorectal cancer. Gut Liver. 2022;16(1):53‐61. 34312323 10.5009/gnl20266 PMC8761926

[32] Fearon K , Strasser F , Anker SD , et al. Definition and classification of cancer cachexia: an international consensus. Lancet Oncol. 2011;12(5):489‐495. 21296615 10.1016/S1470-2045(10)70218-7

[33] Nishigori T , Tsunoda S , Okabe H , et al. Impact of sarcopenic obesity on surgical site infection after laparoscopic total gastrectomy. Ann Surg Oncol. 2016;23(Suppl 4):524‐531. 27380646 10.1245/s10434-016-5385-y

[34] Fleming CA , O'Connell EP , Kavanagh RG , et al. Body composition, inflammation, and 5‐year outcomes in colon cancer. JAMA Netw Open. 2021;4(8):e2115274. 34459908 10.1001/jamanetworkopen.2021.15274 PMC8406082

[35] van der Schroeff MP , Baatenburg DJR . Staging and prognosis in head and neck cancer. Oral Oncol. 2009;45(4–5):356‐360. 18715810 10.1016/j.oraloncology.2008.05.022

[36] Woodard GA , Jones KD , Jablons DM . Lung cancer staging and prognosis. Cancer Treat Res. 2016;170:47‐75. 27535389 10.1007/978-3-319-40389-2_3

